# Colloid Copper in Cancer

**Published:** 1913-11

**Authors:** 


Colloid Copper In Cancer. Leo Loev, C. B., McClurg and W. O. Sweek, *Interstate Med. Jour.,* December, 1912 and January, 1913, report cases treated by daily intravenous injections of 300-400 c.c. of solution. Symptomatic relief and even retrogression were noted. We have, in two inoperable cases, used organic silver salts by high-frequency cataphoresis, with apparent benefit, though one patient died from hepatic metastases after three years of fair general health, and the other was practically moribund and died in a few weeks. In this latter case the tumor broke down and discharged like an abscess, corresponding to the findings of the authors quoted that secretion was increased from exposed cancers.

